# Multiple-data-based monthly geopotential model set LDCmgm90

**DOI:** 10.1038/s41597-019-0239-7

**Published:** 2019-10-23

**Authors:** Wei Chen, Jiesi Luo, Jim Ray, Nan Yu, Jian Cheng Li

**Affiliations:** 1 Collaborative Innovation Center of Geospatial Technology/Key Laboratory of Geospace Environment and Geodesy, School of Geodesy and Geomatics, Wuhan University, Wuhan, China; 2 Retired: National Oceanic and Atmospheric Administration, Silver Spring, Maryland, USA; 3 College of Marine Science and Technology, China University of Geosciences, Wuhan, China

**Keywords:** Geophysics, Climate and Earth system modelling, Hydrology

## Abstract

While the GRACE (Gravity Recovery and Climate Experiment) satellite mission is of great significance in understanding various branches of Earth sciences, the quality of GRACE monthly products can be unsatisfactory due to strong longitudinal stripe-pattern errors and other flaws. Based on corrected GRACE Mascon (mass concentration) gridded mass transport time series and updated LDCgam (Least Difference Combination global angular momenta) data, we present a new set of monthly gravity models called LDCmgm90, in the form of Stokes coefficients with order and degree both up to 90. The LDCgam inputs are developed by assimilating degree-2 Stokes coefficients from various versions of GRACE (including Mascon products) and SLR (Satellite Laser Ranging) monthly gravity data into combinations of outputs from various global atmospheric, oceanic, and hydrological circulation models, under the constraints of accurately measured Earth orientation parameters in the Least Difference Combination (LDC) scheme. Taking advantages of the relative strengths of the various input solutions, the LDCmgm90 is free of stripes and some other flaws of classical GRACE products.

## Abstract (summary)

**Table:** 

Measurement(s)	Stokes coefficient • Coefficient
Technology Type(s)	spherical harmonic expansion • least difference combination • computational modeling technique
Sample Characteristic - Environment	climate system
Sample Characteristic - Location	Earth (planet)

Machine-accessible metadata file describing the reported data: 10.6084/m9.figshare.9924929

## Background & Summary

Time-dependent gravity from the GRACE (Gravity Recovery and Climate Experiment) twin satellites is of great significance for studies related to changes in land water, ice sheets, sea level rise, ocean circulation, Earthquake dynamics etc.^1–7^. GRACE data are routinely provided almost every month (from Apr. 2002 to Jun. 2017, but with 20 months missing) in the form of Stokes coefficients with AOD1B (Atmosphere and Ocean Dealiasing Level 1B) corrections (denoted as GSM) by Center for Space Research (CSR), Deutsches GeoForschungsZentrum (GFZ), Jet Propulsion Laboratory (JPL) and Graz University of Technology (TUG), using a least squares adjustment (LSA) scheme^8–14^. There are often limited agreements between GRACE-based results and those obtained by independent observations, mostly attributed to the well-known strong striped noise patterns caused by the GRACE’s near-polar orbital inclination and the LSA scheme, which ignores the orthogonality of spherical harmonics and thus leads to correlations of Stokes coefficients^15–19^. Notable discrepancies can also be found among GRACE products released by different institutes, due to some differences in data processing strategies adopted by them^8–13,19–22^. Various filtering and destriping methods are proposed to attenuate these stripes, resulting in weaker and distorted signals of interest^23–28^. Moreover, power losses are also found around 3 cycles per year (cpy) and higher in time series of low degree GRACE Stokes coefficients^21^.

Since 2015, CSR and JPL also provide so-called Mascon solutions using the Mass Concentration blocks (mascons)^29–32^, another form of gravity field basis functions. With mascons, some a priori geophysical constraints can be implemented to remove noises from the GRACE observations at the Level-2 processing step, which is a much more rigorous approach than the empirical post-processing filtering and destriping of the LSA-based spherical harmonics. However, the problem of power losses around 3 cpy and higher is not overcome, and notable differences between CSR and JPL mascon solutions still exist (noted by this study).

Mass redistributions will cause changes not only in gravity but also in Earth’s pole coordinates and spin rate, due to conservation of angular momentum^33,34^. Plenty of studies have explored the links between the time-dependent Stokes coefficients and Earth rotational variations, specifically the level of agreement between GRACE-based (*C*_21_, *S*_21_) series and polar motion, and between SLR-based C_20_ and length-of-day (LOD) variations after contributions unrelated to mass redistributions are excluded^21,35–45^. Some even made use of these GRACE and/or SLR (Satellite Laser Ranging) coefficient series to improve geophysically based fluid model excitations of polar motion and LOD variations^21,22,46^. Among these studies, the Least Difference Combination (LDC) of global angular momenta for surficial geophysical fluids of Chen *et al*.^21^ and Yu *et al*.^22^ (hereafter termed as LDCgam) seem to have the best performances in both the frequency and time domains, since various versions (CSR, GFZ and JPL) of GRACE and SLR monthly gravity data (RL05) were assimilated into the outputs from various global atmospheric, oceanic, and hydrological circulation models, in the LDC scheme which can extract the best frequency components from various types of data sources provided that one or more proper reference data or models can be established^21,47^.

To summarize, the currently available GRACE monthly Stokes coefficients are unsatisfactory due to strong longitudinal stripe-pattern errors and other flaws while assimilating independent related observations may help to improve them. In this study, we used numerical integration to convert Mascon gridded mass to Stokes coefficients and applied necessary corrections as described in the next section. We also prepared for this study an updated LDCgam solution^48^ obtained by similar procedures in Chen *et al*.^21^ and Yu *et al*.^22^ but assimilating all RL05 and RL06 GRACE/SLR Stokes coefficients from CSR, GFZ, JPL and TUG, and all RL05 and RL06 Mascon gridded mass fields. Then we put forward the improved monthly gravity model set LDCmgm90, in the form of Stokes coefficients (complete from degree and order 2 to 90) since they are more convenient to use.

## Methods

The GRACE monthly data are usually released together with the GRACE AOD1B products, which provide a model-based data-set (including GAA, GAB, GAC and GAD) that describes the time variations of the gravity potential at satellite altitudes that are caused by non-tidal mass variability in the atmosphere and oceans^49–51^. The GAA product describes the monthly non-tidal atmospheric mass anomalies simulated by the operational run of the atmosphere model ECMWF (European Centre for Medium-Range Weather Forecasts)^52^, GAB refers to monthly non-tidal oceanic mass anomalies simulated by the operational run of the (unconstrained) ocean model OMCT (Ocean Model for Circulation and Tides)^53^ (for RL05) or MPIOM (Max-Planck-Institute for Meteorology Ocean Model)^54^ (for RL06), GAC is the sum of GAA and GAB, and GAD can be regarded as a revised version of GAC with non-tidal atmospheric and oceanic mass anomalies only over ocean areas. GSM is just the gravity residual after GAA and GAB are removed from the GRACE observations (in other words, GSM + GAB + GAA is what GRACE satellites really measure). Consistent with this system, the LDCmgm90 data set also contains five subsets GAA, GAB, GAC, GAD and GSM, all with degree and order up to 90 because higher harmonics are not guaranteed by GRACE’s measurement resolution.

**Fig. 1:**
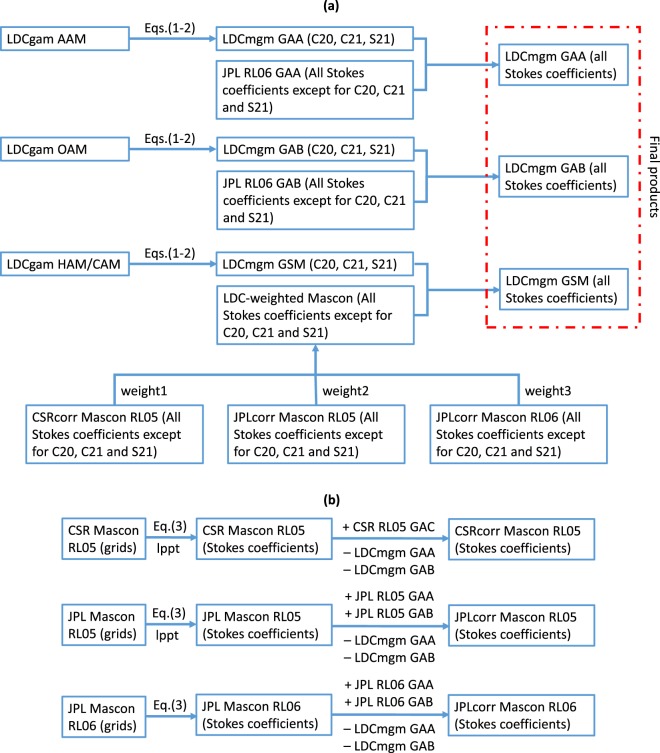
Flow chart describing the procedures to generate the monthly global gravity model series LDCmgm90. (**a**) General procedures. (**b**) Procedures to obtain and correct Stokes coefficients derived from different Mascon solutions. The weights are determined by Eq. (4) and lppt = long-period pole tide correction according to Wahr *et al*.^20^.

The general procedures to produce the LDCmgm90 are described in Fig. 1, which is explained next.

### Step 1: Obtain the LDCmgm degree-2 zonal and tesseral potential coefficients

We first obtained elements of the inertia tensor (Δ*I*_*xz*_(*t*), Δ*I*_*yz*_(*t*), Δ*I*_*zz*_(*t*)) through the mass-redistribution-related (or mass-term) angular momenta LDCgam:

1 $${{\bf{h}}}^{LDC}\equiv ({h}_{x}^{LDC},\,{h}_{y}^{LDC},\,{h}_{z}^{LDC})=\Omega (\Delta {I}_{xz}^{LDC}(t),\,\Delta {I}_{yz}^{LDC}(t),\,\Delta {I}_{zz}^{LDC}(t)),$$

then the corresponding LDCmgm degree-2 zonal and tesseral potential coefficients^31,52^

2 $$\begin{array}{lll}\Delta {C}_{20}^{LDC}(t) & = & \frac{1}{2\sqrt{5}}\frac{1+k{^{\prime} }_{2}}{M{a}^{2}}\left[\Delta T-3\Delta {I}_{zz}^{LDC}(t)\right],\\ \Delta {C}_{21}^{LDC}(t) & = & -\sqrt{\frac{3}{5}}\frac{1+k{^{\prime} }_{2}}{M{a}^{2}}\Delta {I}_{xz}^{LDC}(t),\\ \Delta {S}_{21}^{LDC}(t) & = & -\sqrt{\frac{3}{5}}\frac{1+k{^{\prime} }_{2}}{M{a}^{2}}\Delta {I}_{yz}^{LDC}(t).\end{array}$$

In Eqs (1) and (2), Ω = 7.292115 10^−5^ rad/s is the mean spin rate of the Earth, *k*′ = −0.316 is the degree-2 load Love number^33^, *M* = 5.97236 × 10^24^ kg and *a* = 6378136.6 m are the mass and mean equatorial radius of the Earth^55,56^, respectively, Δ*T* is the change in the trace of the inertia tensor and equals zero in the current case that the global mass is conserved^33,57^.

The LDCgam provides atmospheric angular momentum (AAM), oceanic angular momentum (OAM) and hydrological angular momentum/cryospheric angular momentum (HAM/CAM), where the HAM/CAM is dominated by but not limited to changes in land water and ice, since all the non-atmospheric and non-oceanic mass redistributions are attributed to it. Therefore, we have the following links (→ means corresponding to):

Mass-term AAM → GAA *C*_20_, *C*_21_ and *S*_21_.

Mass-term OAM → GAB *C*_20_, *C*_21_ and *S*_21_.

Mass-term HAM/CAM → GSM *C*_20_, *C*_21_ and *S*_21_.

Then we can obtain the degree-2 GAA, GAB and GSM zonal and tesseral potential coefficients for the LDCmgm90 (please refer to the top half of Fig. 1a). Noting that CSR, GFZ, JPL and TUG all used the same AOD1B products for the given data releases (RL05 or RL06), and the JPL GAA, GAB, GAC and GAD products are the most complete, we thus chose the JPL RL06 GAA, GAB, GAC and GAD products to construct the LDCmgm90.

### Step 2: Convert the Mascon gridded mass redistribution to corrected Stokes coefficients

Currently, there are three Mascon solutions CSR Mascon RL05, JPL Mascon RL05 and JPL Mascon RL06, of which the original Mascon gridded data correspond to the GSM products^29–31^. Although the RL05 and RL06 Mascon products are based on different static background geopotential model (which would cause biases among them), we are more interested in the time-dependent parts rather than the static ones when using GRACE-like products. With these biases removed, a proper combination can extract the best components from these three Mascon solutions since no original single solution is perfect as discussed in Background & Summary.

The Mascon data are represented in the form of equivalent water height Δ*h*(*θ*, *λ*, *t*) on a 0.5 degree longitude-latitude grid but representing the equal-area geodesic grid of size 1 × 1 degree at the equator. The surface density for this thin layer is Δ*σ*(*θ*, *λ*, *t*) = *ρ*_*w*_Δ*h*(*θ*, *λ*, *t*), where *ρ*_*w*_ = 1025 kg/m^3^ is the average density of sea water. Then the original Mascon gridded data may be converted to Stokes coefficients by^58^

3 $${\left\{\begin{array}{c}\Delta {C}_{nm}(t)\\ \Delta {S}_{nm}(t)\end{array}\right\}}_{surfacemass}=\frac{3}{4\pi }\frac{1+k{^{\prime} }_{n}}{a{\rho }_{ave}(2n+1)}\int \Delta \sigma (\theta ,\lambda ,t){P}_{nm}(cos\theta )\left\{\begin{array}{c}cos(m\lambda )\\ sin(m\lambda )\end{array}\right\}\,sin\,\theta d\theta d\lambda ,$$

where $k{^{\prime} }_{n}$ is the degree-n load Love number (from Table 1 of Wahr *et al*.^58^), *ρ*_*ave*_ = 5517 kg/m^3^ is the average density of the solid Earth.

**Table 1:** Data used.

Data	Data access
CSR GRACE RL05 GSM /GAC/GAD* ^61^	ftp://isdcftp.gfz-potsdam.de/grace/^#^
CSR GRACE RL06 GSM/GAC/GAD* ^62^	ftp://isdcftp.gfz-potsdam.de/grace/
CSR GRACE Mascon RL05 (Version 1)^31^	http://www2.csr.utexas.edu/grace
CSR SLR RL05^63–65^	ftp://ftp.csr.utexas.edu/
CSR SLR RL06^63–65^	ftp://ftp.csr.utexas.edu/
GFZ GRACE RL05 GSM/GAA/GAB/GAC/GAD^66^	ftp://isdcftp.gfz-potsdam.de/grace/^#^
GFZ GRACE RL06 GSM/GAA/GAB/GAC/GAD^67^	ftp://isdcftp.gfz-potsdam.de/grace/
JPL GRACE RL05 GSM/GAA/GAB/GAC/GAD^68^	ftp://isdcftp.gfz-potsdam.de/grace/^#^
JPL GRACE RL06 GSM/GAA/GAB/GAC/GAD^69^	ftp://isdcftp.gfz-potsdam.de/grace/
JPL GRACE Mascon RL05M (Version 2)^29,30^	http://grace.jpl.nasa.gov
JPL GRACE Mascon RL06 (Version 1)^32^	http://grace.jpl.nasa.gov
LDCgam^20,21,48^	https://doi.org/10.13140/RG.2.2.28698.49604
TUG ITSG-Grace2018 RL06^14^	https://doi.org/10.5880/ICGEM.2018.003

*GAA and GAB are not provided by CSR.

^#^No longer available since they ceased to provide the RL05 solutions very recently.

The GAA RL05 produced by the ECMWF operational run contains the following two notable jumps^49,59^:

1. Between 2006-01-29 18 h and 2006-01-30 00 h
2. Between 2010-01-26 00 h and 2010-01-26 06 h

due to upgrades of the horizontal and vertical resolutions in the ECMWF model, which will lead to opposite jumps in all the corresponding RL05 versions of GSM and Mascon products. Moreover, the RL05 products adopted the non-linear IERS2010 mean pole correction^56^, which will cause a long-period pole tide in *C*_21_ and *S*_21_ and should be corrected as suggested by Wahr *et al*.^20^. For the two RL05 Mascon products, corrections of the jumps and long-period pole tide should be applied (see Fig. 1b) while the RL06 data are free of these flaws due to a homogeneous reanalysis of the ECMWF data and the adoption of a linear mean pole model. However, one must keep in mind that whichever RL05 or RL06, GAA and GAB are respectively derived from the ECMWF and OMCT (or MPIOM) operational outputs, which need further refinements as shown in detailed analyses by Chen *et al*.^21,47^ and Yu *et al*.^22^. Thus it would be better to replace them with the LDC-corrected GAA and GAB. Further, GAC = GAA + GAB, and GAD can also be obtained by applying an ocean mask to GAC.

By using Eq. (3) and applying the above-mentioned corrections and replacements, we can obtain the corrected Mascon Stokes coefficients as shown in Fig. 1b.

### Step 3: Take weighted average of the corrected Mascon Stokes coefficients and obtain the final solutions

The GRACE-observed geopotential *V*_obs_ may be separated into two parts: the part ${V}_{obs}^{zt}$ including the degree-2 zonal and tesseral terms (namely the terms relevant with *C*_20_, *C*_21_ and *S*_21_), and the other ${V}_{obs}^{nzt}$ containing all other terms, namely ${V}_{obs}={V}_{obs}^{zt}+{V}_{obs}^{nzt}$. All the CSR, GFZ, JPL and TUG released GRACE data are from the same twin satellites, thus in principle, any overestimate or underestimate of ${V}_{obs}^{zt}$ will cause an opposite effect on ${V}_{obs}^{nzt}$. That is, ${V}_{obs}^{zt}$ and ${V}_{obs}^{nzt}$ must have the same errors for each given version of GRACE data. Based on this reasoning, the weights of the corrected Mascon Stokes coefficients may be estimated as

4 $$\begin{array}{l}1/weight\,1={std}^{2}({C}_{20}^{CSRcorr05}-{C}_{20}^{LDCmgm})+{std}^{2}({C}_{21}^{CSRcorr05}-{C}_{21}^{LDCmgm})+{std}^{2}({S}_{21}^{CSRcorr05}-{S}_{21}^{LDCmgm})\\ 1/weight\,2={std}^{2}({C}_{20}^{JPLcorr05}-{C}_{20}^{LDCmgm})+{std}^{2}({C}_{21}^{JPLcorr05}-{C}_{21}^{LDCmgm})+{std}^{2}({S}_{21}^{JPLcorr05}-{S}_{21}^{LDCmgm})\\ 1/weight\,3={std}^{2}({C}_{20}^{JPLcorr06}-{C}_{20}^{LDCmgm})+{std}^{2}({C}_{21}^{JPLcorr06}-{C}_{21}^{LDCmgm})+{std}^{2}({S}_{21}^{JPLcorr06}-{S}_{21}^{LDCmgm})\end{array}$$

since C_20_, C_21_ and S_21_ obtained from LDCgam are the most accurate and may be approximately used as standards to infer errors in other data sets. In Eq. (4), std(*x*) means standard derivation of *x*. The corresponding relative weights of the three Mascon solutions can be found in Table 3b.

We can obtain the weighted average of the corrected Mascon Stokes coefficients except for *C*_20_, *C*_21_ and *S*_21_ as described in the bottom part of Fig. 1b.

## Data Records

Availabilities of the data used in this study are summarized in Table 1. While most GRACE and SLR data sets are named after their releasing institutes, the latest GRACE data set computed at TUG is termed ITSG-Grace2018 (ITSG for short). Data after Aug. 2016 (7 data points in total) are not provided by all RL06 GRACE products, and are supplemented by the corresponding RL05 ones.

The LDCmgm90 dataset is provided in the netcdf 4.0 format and can be accessed via figshare^60^, which contains five subsets GAA, GAB, GAC, GAD and GSM, all in the form of Stokes coefficients complete from degree and order 2 to 90.

## Technical Validation

**Fig. 2:**
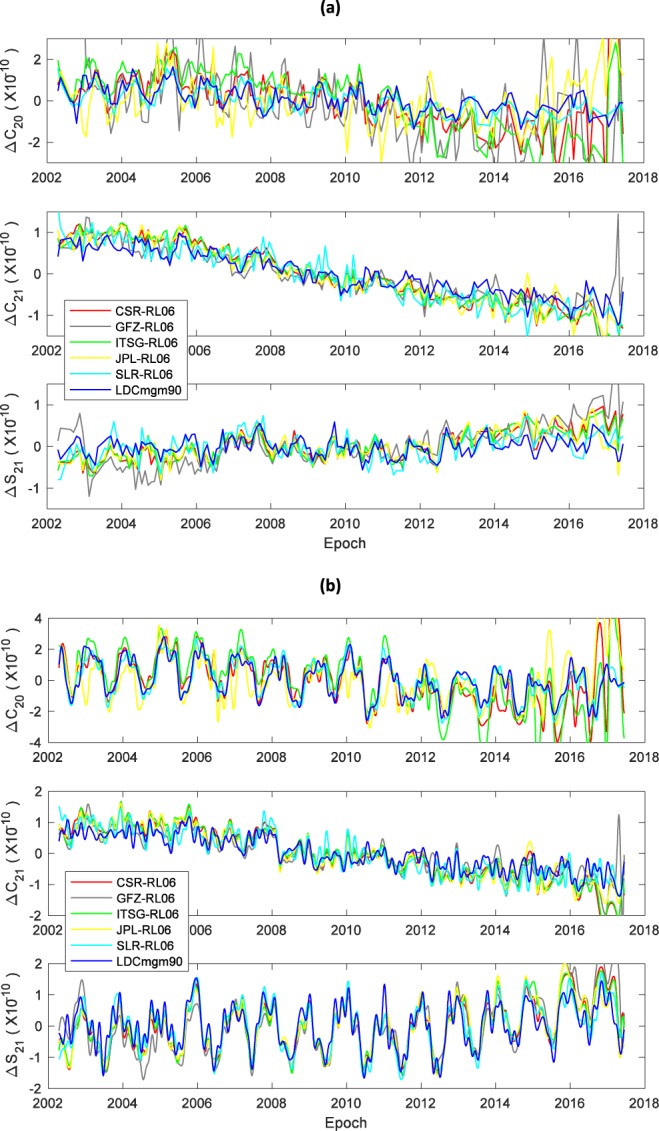
*C*_20_, *C*_21_ and *S*_21_ series from different sources. (**a**) GSM data for 163 months (no interpolation applied); (**b**) GSM + GAA + GAB data with cubic spline interpolations for better displays of seasonal cycles. The means of all series are removed.

**Fig. 3:**
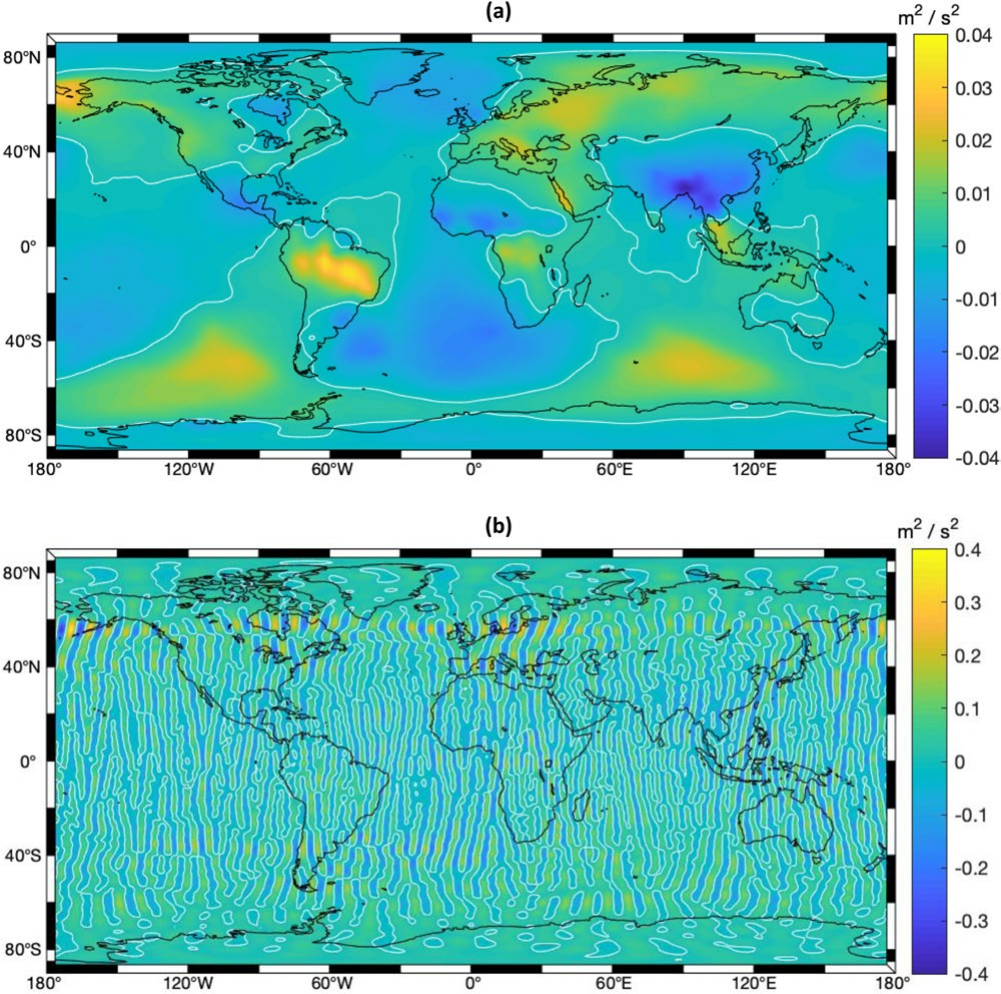
Differences between the geopotential maps for Nov 2010 and Oct 2010. (**a**) Results from LDCmgm90; (**b**) Results from CSR RL06. Neither smoothing nor destriping is applied to either figure.

The degree-2 GSM zonal and tesseral Stokes coefficients from LDCmgm90 and other individual releases are compared in Fig. 2a, while the GSM + GAA + GAB ones are compared in Fig. 2b. One can see the coefficients from LDCmgm are less noisy and free of anomalous signals presented in some other GRACE products, since when combining or assimilating data from different sources, the LDC method can provide a good handle of both the magnitude and phase aspects simultaneously for arbitrary frequency including the lowest frequency component which is usually called the trend of a series^21,47^. The standard derivations of the original and corrected LDCmgm GSM (*C*_20_, *C*_21_, *S*_21_) with respect with those from other model sets are provided in Tables 2 and 3, respectively. In addition, Figs 1, 3, 4 and Table 4 of Chen *et al*.^21^ implied that our *C*_21_ and *S*_21_ (the corresponding geophysical excitations are denoted as LDCgsc) are the most consistent with the observed polar motion, while Fig. 9 and Table 5 of Yu *et al*.^22^ suggested our *C*_20_ agrees the best with the observed length-of-day variations. A further and more independent check of the LDCmgm90 would be to compute the loads from the monthly gravity fields and apply those to GPS time series. However, the complexity of such a check makes it impossible to include in this short data descriptor so that will left for later work.

**Table 2:** Statistical information for original GSM data (unit: 10^−11^).

X	${\bf{std}}\,({{\boldsymbol{C}}}_{{\bf{20}}}^{{\bf{X}}}-{{\boldsymbol{C}}}_{{\bf{20}}}^{{\bf{LDC}}})$	${\bf{std}}\,({{\boldsymbol{C}}}_{{\bf{21}}}^{{\bf{X}}}-{{\boldsymbol{C}}}_{{\bf{21}}}^{{\bf{LDC}}})$	${\bf{std}}\,({{\boldsymbol{S}}}_{{\bf{21}}}^{{\bf{X}}}-{{\boldsymbol{S}}}_{{\bf{21}}}^{{\bf{LDC}}})$
CSR RL05 Mascon	9.456	7.121	2.981
JPL RL05 Mascon	9.626	5.471	4.903
JPL RL06 Mascon	9.950	2.588	3.602
CSR RL05	16.859	6.231	2.497
GFZ RL05	49.399	3.756	3.606
JPL RL05	15.078	5.520	3.971
CSR RL06	11.134	3.429	3.021
GFZ RL06	23.359	3.637	5.121
ITSG RL06	14.911	3.378	3.047
JPL RL06	11.222	3.054	2.934
SLR RL05	5.518	5.700	2.655
SLR RL06	5.250	3.190	2.536

**Table 3:** Statistical information (unit: 10^−11^) and relative weights for corrected Mascon GSM data.

X	${\bf{std}}\,({{\boldsymbol{C}}}_{{\bf{20}}}^{{\bf{X}}}-{{\boldsymbol{C}}}_{{\bf{20}}}^{{\bf{LDC}}})$	${\bf{std}}\,({{\boldsymbol{C}}}_{{\bf{21}}}^{{\bf{X}}}-{{\boldsymbol{C}}}_{{\bf{21}}}^{{\bf{LDC}}})$	${\bf{std}}\,({{\boldsymbol{S}}}_{{\bf{21}}}^{{\bf{X}}}-{{\boldsymbol{S}}}_{{\bf{21}}}^{{\bf{LDC}}})$	Relative weight
CSR RL05 Mascon	7.908	6.568	5.053	0.2938
JPL RL05 Mascon	8.051	5.102	6.404	0.2924
JPL RL06 Mascon	8.222	3.003	4.068	0.4138

The mutual differences of geopotential maps for two neighboring months are also compared in Fig. 3. One can see the one corresponding to LDCmgm90 has no stripes, thanks to the Mason solutions used, while those for CSR, GFZ, JPL and TUG (only the map for CSR RL06 is provided here) have strong stripe-pattern noises, which overwhelm any geophysical signal of interest.

## Data Availability

The MatLab codes used to generate the LDCmgm90 are available upon request to W. Chen (wchen@sgg.whu.edu.cn).
